# The Choice of Surgical Specialization by Medical Students and Their Syncopal History

**DOI:** 10.1371/journal.pone.0055236

**Published:** 2013-01-31

**Authors:** Jerzy Rudnicki, Dorota Zyśko, Dariusz Kozłowski, Wiktor Kuliczkowski, Edward Koźluk, Małgorzata Lelonek, Agnieszka Piątkowska, Jacek Gajek, Marta Negrusz-Kawecka, Anil Kumar Agrawal

**Affiliations:** 1 Department of Minimally Invasive Surgery and Proctology, Wroclaw Medical University, Wrocław, Poland; 2 Teaching Department for Emergency Medical Services, Wroclaw Medical University, Wrocław, Poland; 3 Department of Cardiology, Medical University of Gdańsk, Gdańsk, Poland; 4 1st Department of Cardiology, Medical University of Warsaw, Warsaw, Poland; 5 Department of Cardiology, Warsaw Medical University, Warsaw, Poland; 6 Department of Cardiology, Medical University of Lodz, Lodz, Poland; 7 Department of Cardiology, Wroclaw Medical University, Wrocław, Poland; 8 2nd Department of General and Oncological Surgery, Wroclaw Medical University, Wrocław, Poland; Sapienza University of Rome, Italy

## Abstract

**Background:**

The aim of the study was to assess whether medical students’ fainting outside the university or while witnessing surgical procedures and/or autopsies influenced their choice of a specialization.

**Materials and Methods:**

The study group consisted of 605 medical students (from fourth to sixth year of study) from five medical universities in Poland (325 women, 212 men and 8 responders of an unspecified gender). The median age of subjects studied was 23 years, and the interquartile range was 23–24 years. The students at each university were chosen randomly by the author who worked there and had contact with them. An anonymous questionnaire was developed to gather information regarding demographics, the specialization which each student wanted to choose, the syncope occurrence in the medical history, the syncope and presyncope occurrence during surgery and autopsy as well as the syncopal events’ characteristics.

**Results:**

The group of 15% of women and 30% of men declared to have pursued the surgical specialization (P<0.001), 29% of women and 56% of men declared the intention to pursue an invasive specialization (P<0.001). As many as 36.0% of women studied and 13.1% of men studied reported syncopal spells outside university (P<0.001). Only 41 students (6.8%) reported that syncope or presyncope in any studied circumstances had an impact on their specialization choice. The multivariate analysis showed that the choice of surgical specialization is related to the male gender and the absence of syncopal spells outside the university.

**Conclusions:**

Syncopal and presyncopal spells may affect the professional choices of the medical students. The male gender and a lack of syncope occurrence outside operating room are related to the choice of surgical specialization.

## Introduction

The lifetime incidence of vasovagal syncope in the general population is about 30% [1]–[5]. The syncopal episodes decrease the quality of life, expose the subject to injuries caused by falls and might discourage from pursuing careers in an environment which exposes the subjects to prolonged orthostatic stress, blood, invasive medical procedures as well as a responsibility for the other people’s lives [6]–[8].

The choice of specialization made by medical students is their own decision which, however, could often be influenced by gender, family tradition, not to mention local factors influencing the opportunity to take the specialization and a current demand on the labour market [9]–[12].

The medical specializations are divided into surgical/invasive and internal/non-invasive ones. In the surgical specializations a diagnosis and a treatment is mainly achieved by surgical techniques. There are also certain special areas in internal medicine implementing surgical procedures, like invasive cardiologists implanting pacemakers working more like surgeons than internal physicians. Currently, division between specializations should rather be made on invasive treatment involved.

So far, there are no formal guidance or precise recommendations developed advising medical students on the choice of specialization. However, it was demonstrated that syncope outside the operating room increases the risk of syncope and presyncope during surgery, although their incidence is low [13].

The purpose:

The aim of the study was to assess the influence of syncopal spells on restriction of professional choices in medical students, with regard to choice of the specialization. Syncopal spells taken into consideration took place in the daily life circumstances (outside the university) or while performing surgical procedures and viewing autopsies.

## Materials and Methods

The study group consisted of 605 medical students (fourth to sixth year of study) from five medical universities in Poland (325 women, 212 men and 8 responders of an unspecified gender). The median age of studied subjects was 23 years, and the interquartile range was 23–24 years. The students at each university were chosen on a random basis by the author who worked there and had contact with them.

An anonymous questionnaire was developed to gather information regarding demographics, the specialization which each student wanted to pursue, the syncope occurrence in the medical history, the syncope and presyncope occurrence during surgery and autopsy as well as the syncopal events’ characteristics, the medical history of supraventricular tachycardia, diabetes, epilepsy, bifascicular block, and asystole.

The list of medical specializations was derived from the official website of the Medical Centre of Postgraduate Education and consisted of 39 items, the so-called basic specializations. General surgery, orthopaedics and trauma surgery, cardiac surgery, gynaecology and obstetrics, paediatric surgery, thoracic surgery, neurosurgery, urology, plastic surgery, maxillofacial surgery were considered the surgical specializations.

In addition to all the surgical specializations, the following specializations were considered invasive: anaesthesiology and intensive care, emergency medicine, laryngology and ophthalmology. Cardiology was considered a specialization related to the invasive treatment if the student selected that preferred invasive specialization.

If a student was unable to choose a specialization he was asked to determine whether he would prefer specializations related to invasive procedures or to non-invasive treatment.

When a student marked two or more specializations and at least one of them was a surgical one it was recorded that he chose the surgical specialization. Similarly, when he selected at least one invasive specialization it was noted that he chose an invasive specialization.

The students who reported syncopal or presyncopal spells either outside or inside their university were asked to determine whether that fact had an impact on their decision regarding the specialization they wished to pursue (perceived impact).

The study was approved by the Commission of Bioethics at Wroclaw Medical University (No. 188/2012).*Statistical analysis.*

The continuous variables were presented as a mean and standard deviation, the comparisons were performed with ANOVA. The categorical variables were presented as numbers and percentages. The comparisons were performed with the chi-square test.

Age as a variable was dichotomised for the statistical purposes, based on the student’s median age, in two groups: 23 years of age and younger, as well as older than 23 years. The number of syncopal spells was dichotomised according to its median number to at least 2 spells and more than 2 spells.

Classification and Regression Trees (CART) analysis was performed to identify factors associated with the choice of surgical and invasive specializations and age (dichotomous at median age), gender, syncopal history, median number of syncopal spells.

CART analysis was performed to find out whether the syncopal history is related to the choice of a surgical specialization beyond the perceived impact of the syncopal history on the choice of a specialization and to assess which syncopal spells: outside or inside the university, are more important regarding the choice of the specialization.

Logistic regression analysis was performed to find an association between the choice of surgical specialization and age (dichotomous at median age), gender, syncopal history, median number of syncopal spells.

CART is a non-parametric method of identifying predictor variables by using binary partitioning: subsets of patients which are formed by examining each possible cut point of each variable to identify the cut point that resulted in the maximum discrimination between subgroups of patients with respect to the probability of an assessed outcome. CART generates a classification rule which can be visualized as a “classification-tree”.

The CART analysis is a non-linear multivariable method to analyse the relationship among an assessed outcome and independent variables. It is of special value if the presumed factors which are subjected to an assessment may have a different relation to the assessed outcome in different subgroups or if such relation exists only in a subgroup what makes the relation difficult to be elucidated by the linear methods. The direction of the relation may differ in the subgroups, for example the voice and the appeal: In men the high voice may be related to their lower attractiveness but in women it it may be related to higher attractiveness. The linear methods (a logistic regression) in such a case would find no relation between an attractiveness and a pitch of voice.

The CART analysis result is presented in a form of a graph. The graph consists of rectangles (called ‘nodes’) connected with lines. The rectangles are numbered, and a number distinctive for each rectangle is positioned in the left upper corner. The first node is called ‘root node’. The rectangle which is split into two rectangles is also often called ‘parent node’ whereas two rectangles growing up from the parent node are called ‘child nodes’. The nodes which are not split into another 2 nodes and are marked by a red edge are called ‘leaves’ (the graph represents a tree turned upside down). If a rectangle is split two lines from its bottom are drawn and they are connected to another rectangles. The text below the node describes the split. The numbers above the lines represent the number of cases directed into the child nodes.

The bars in each rectangle represent cases. The legend identifying what bars in the node histograms correspond to the assessed outcome is usually located in the top-left corner of the graph.

The proportion between the rectangles’ heights represents the proportion between the number of cases with and without the assessed outcome The leaf represents the group of patients who met the criteria from the first node (root) to the given leaf, and the number in the right corner denotes a predicted assessed outcome of that subgroup of patients. In the CART analysis a set of rules is developed for dividing a large heterogeneous population into smaller, more homogeneous groups with a respect to a particular target variable. The sensitivity, the specificity of constructed models as well the accuracy of a global 10-fold cost validation of the CARTs were presented.

P values less than 0.05 were considered significant.

## Results

The studied group consisted of medical students who were generally healthy. Five students reported supraventricular tachycardia in their medical history: 2 out of them had syncopal events outside the university and 3 had no syncope outside the university. Two of those with supraventricular tachycardia had minor presyncopal complaints during a surgery and no syncopal or presyncopal events during autopsy.

One student reported epilepsy and had no syncopal or presyncopal event during surgery or autopsy but reported events of loss of consciousness outside the university. No student reported diabetes, bifascicular block or asystole.

### Choice of the Surgical and Invasive Specialization

The group of 15% of women and 30% of men declared choice of the surgical specialization, the difference being statistically significant (P<0.001). Furthermore, 29% of women and 56% of men declared the choice of invasive specialization (P<0.001).

#### Syncopal and presyncopal events

A total of 167 medical students studied (27.6% of the study group) had reported the syncopal episodes outside the university, 8 (1.28%) students had syncope during the surgery and one man (0.16%) – during the autopsy.

The prevalence of the syncopal spells in daily life circumstances (outside the university) was much higher in women (36%) than in men (13.1%), the difference being statistically significant (P<0.001). Presyncopal events outside the university forcing studied subjects to stop their current activity were also more prevalent in women than in men (46.9% vs. 17.6% P<0.001). The syncopal events during the surgery were also more prevalent in women than in men (1.9% vs. 0% P = 0.038), Severe presyncopal spells forcing students to leave the surgical table/the operating room were more prevalent in women than in men: 14.7% vs. 4.1% P<0.001, as were mild presyncopal spells allowing for staying at the operating table (16.0% vs. 7.2% P = 0.002).

There were no statistically significant differences in the syncopal and presyncopal events’ occurrence during the autopsy between women and men: syncopal events 0.3% vs. 0% p = 0.41 (p = NS); presyncopal events: 3.7% vs. 2.7% p = 0.51 (p = NS).

The number of syncopal spells did not have a normal distribution. The median number of syncopal spells in the whole syncopal group was 2, and the interquartile range (IQR) was 1–3 syncopal spells. The mean number was 2.6, and its standard deviation was 2.1.

Syncope was reported: at the sight of blood – by 2.9% of women and 1.8% of men (P = NS), at injections – by 2.1% of women and 1.4% of men (P = NS), while presyncope was reported at sight of blood by 7.7% and 3.1% (P<0.05) and at injections – by 7.5% and 7.2% (P = NS) of women and men, respectively.

### Syncopal and Presyncopal Events at Different Sites

In all centres participating in the study the majority of students declared that they watched surgeries in the course of their classes in an operating theatre, however there were statistically significant differences among the frequencies of the involvement in surgical procedures (Table 1).

**Table 1:** The differences of students’ involvement in surgical procedures among the centres.

Medical university	Percentage ofstudents whowatched surgicalprocedures	Percentage ofstudentspartaking inthe surgery	Syncopal spellsin the operatingroom N (%)	Severe presyncopalspells requiring thestudent to leave thesurgical table/operatingroom N (%)	Mild presyncopalspells allowing fora stay at theoperating tableN (%)	Cumulativeincidence ofsyncopal andpresyncopalevents (%)
Wrocław N = 193	100.0	96.4	5 (2,6)	27 (14.0)	29 (15.0)	31.6
Gdańsk N = 213	99.1	63.4	0^$^	22 (10.3)	24 (11.3)	21.6* #
Łódź N = 65	100.0	81.5	0	10 (15.4)	14 (21.5)	36.8
Warsaw N = 99	92.9	61.6	3 (3.0)	6 (6.1)	7 (7.1)	13.1* #
Zabrze N = 35	97,1	94.3	0	0	4 (11.4)	11.4* #
Average	98.4	77.4	8 (1.3)	65 (10.7)	78 (12.9)	25.0

* - P<0.05 vs. Wrocław.

# - P<0.05 vs. Łódź.

98.4% of the studied population reported that they had watched surgical operations. One student reported that he did not watch any surgical operation but he had a mild presyncope in the operating room. Six students did not answer the question about syncope and presyncope in the operating room, four of them did not watch it, and the other two watched the operation (the first participated in the operation, the other did not) and they had no syncope outside the university.

The percentage of students involved in the surgery was significantly higher in Wrocław and Zabrze than in other medical universities. Moreover, the percentage of students with presyncopal and syncopal episodes during surgery was significantly higher in Wrocław than in Zabrze.

#### Syncopal and presyncopal spells during autopsy

602 students answered the question regarding syncope or presyncope during autopsy and 3 (0.5%) did not.

One student (0.16%) reported syncope during autopsy and 22 students (3.6%) reported presyncope during this procedure.

Out of 23 students reporting presyncope or syncope 14 of them had syncope in their medical history outside the university.

The distribution of the incidence of syncopal and presyncopal spells during autopsy among centres studied was presented in Table 2. There were no significant differences between frequency of syncope or presyncope among centres.

**Table 2:** The syncopal and presyncopal spells in the studied medical students population.

Medical university	Syncopal spell at autopsy N (%)	Presyncopal spell at autopsy N (%)
Wrocław	0	12 (6.2)
Gdańsk	0	8 (3.8)
Łódź	0	0
Warsaw	1 (1.0)	1 (1.0)
Zabrze	0	1 (2.9)
Total	1 (0.17)	22 (3.6)

#### Perceived impact of the syncopal and presyncopal spells

41 students (6.8%) reported that syncope or presyncope in any studied scenario had an impact on their specialization choice. Syncopal and presyncopal events in all three situations had an impact on the choice in 5 students, in two situations – in 21 students, and in one situation – in 15 students.

38 students (92.7% out of 41 students who reported that the syncope had an impact on their choice) did not choose the surgical specialization, and 3 students (1 male student with syncope outside university, 1 female student with presyncope outside university, 1 male student with mild presyncope during surgery and presyncope during autopsy), who reported that the syncope had an impact on their choice, did choose the surgical specialization.

The percentages of students with syncopal spells and presyncope outside the university, in operating room or during autopsy, and the percentages of those, who bestowed its influence on specialization choice and not choosing surgery, were shown in Table 3.

**Table 3:** The impact of previous syncopal or presyncopal spells on the choice of the specialization by medical students.

Medical university	Syncopal or presyncopal spells outside university		Syncopal or presyncopal spells in the operating room		Syncopal or presyncopal spells during autopsy	
	Prevalence (%)	Impact on the choice (%)	Prevalence (%)	Impact on the choice (%)	Prevalence (%)	Impact on the choice (%)
Wrocław	50.3	5.7	32.6	4.7	7.3	2.1
Gdańsk	46.0	6.1	22.5	6.1	4.2	0.9
Łódź	55.4	6.2	36.9	7.7	0.0	4,6
Warsaw	44.4	3.0	18.2	4.0	2.0	1.0
Zabrze	14.3	2,9	11.4	2,9	2.9	2.9
Average	46.3	5.5	26.0	5.3	4.3	1.8

#### Multivariate analysis

The choice of surgical specialization.

**Figure 1:**
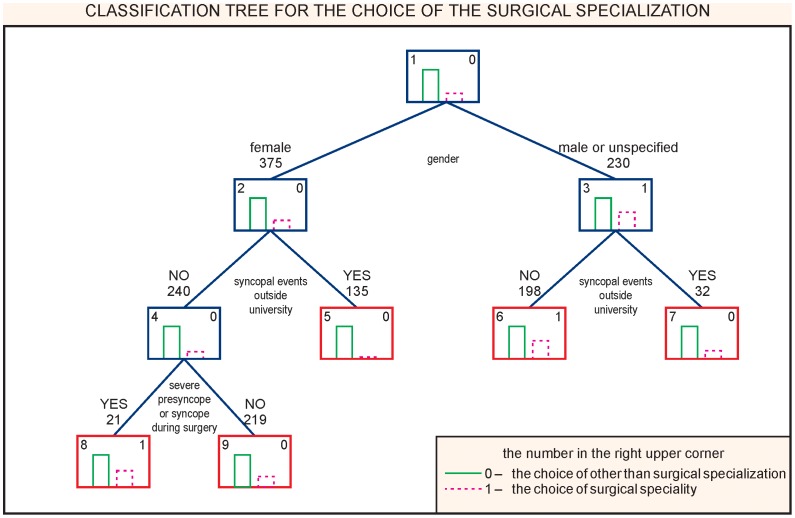
CART analysis. Choice of the surgical specialization.

The choice of surgical specialization is related to the male gender and the absence of syncopal spells outside the university. However, in women it is the presence of presyncopal or syncopal events in the operating room that is related to the choice of surgical specialization. (Figure 1). The sensitivity of the model was 57.4% and the specificity 69.6%; accuracy of 10-fold global cost validation was 0.67.

The logistic regression analysis revealed that only the absence of syncopal spells outside university was related to the choice of a surgical specialization (OR: 2.7; 95% CI 1.6–4.5, P<0.001).

**Figure 2:**
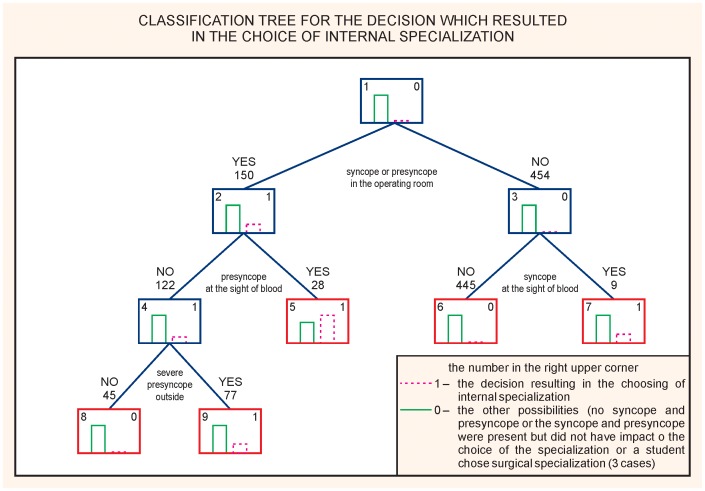
CART analysis. What syncopal events influence the choice of the specialization.

**Figure 3:**
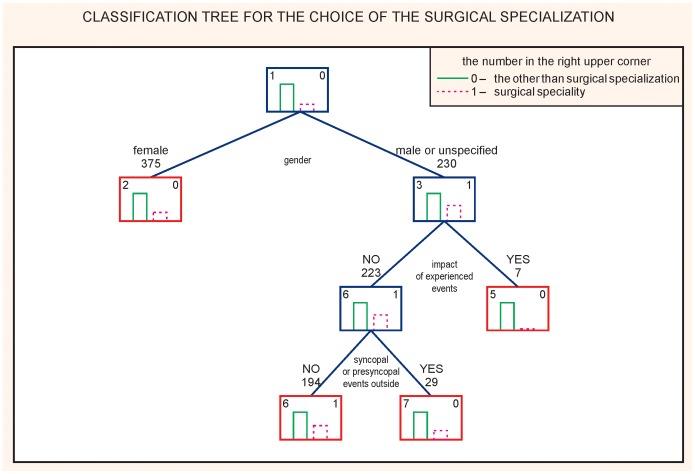
CART analysis. Choice of the surgical specialization (after taking into account the students’ own assessment that an incident of syncope or presyncope had an impact on the choice of the specialization other than surgery).

To the students’ minds, the occurrence of a syncope or a presyncope in an operating room had the most impact on the choice of a specialization. (Figure 2). The sensitivity of the model was 53.1% and the specificity 73.1%; accuracy of global 10-fold global cost validation of the presented CART was 0.56. The next analysis was performed to assess whether syncope or presyncope outside university were related to the choice of specialization regardless of their perceived impact. It was found that the syncope outside university remains a factor influencing the choice of a specialization other than a surgical one. (Figure 3). The sensitivity of the model was 51.1% and the specificity 72.8%; accuracy of a global 10-fold global cost validation of the presented CART was 0.60.

#### The choice of non-invasive specialization

**Figure 4:**
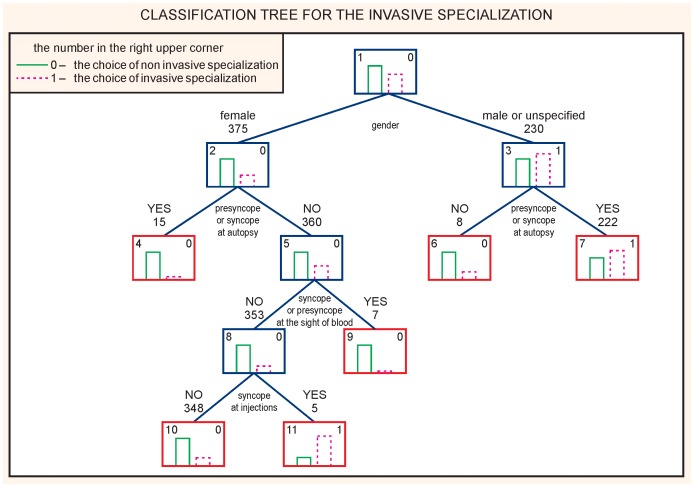
CART analysis. Choice of the invasive specialization.

The study shows that the most important factor related to the choice of the invasive specialization was male gender. In men and women the syncopal or presyncopal episodes at an autopsy were related to the choice of the non-invasive specialization. Additionally, in women a syncope or a presyncope at the sight of blood was related to the choice of a non-invasive specialization (Figure 4). The sensitivity of the model was 53.1% and the specificity 73.1%; accuracy of a global 10-fold global cost validation was 0.62.

The logistic regression analysis revealed that the male gender (OR: 12.8; 95% CI 2.5–65.8) and the absence of syncopal spells in the medical history were related to the choice of the surgical specialization (OR: 1.5; 95% CI 1.02–2.2, p<0.001).

## Discussion

The occurrence of a vasovagal syncope results in the decreased quality of life, due to the threat of possible injuries resulting from the fall. The decrease in the quality of life experiencing by vasovagal patients is similar to those suffering from epilepsy and is related to the episodic, unpredictable, and recurrent nature of the events of a loss of consciousness [8]. The age at the first vasovagal syncope is usually 14 years – just before making the first decisions regarding future profession selection [4]. Choosing the future profession depends on interests and individual mental, intellectual and physical predispositions.

### Syncopal Events Incidence

The first finding of the study was the similar rate of the syncopal events incidence as in the other reports concerning the general population [1], [4]. Moreover, syncopal spells happen twice as often by women than by men what is also a hallmark of a vasovagal syncope. Comparable relations referring to the frequency and the gender were reported in the Polish population of the young adults [5]. This finding indicates that the choice of a medical profession is not influenced by the syncopal events occurrence in the medical history. Similar results were reported by Ganzebon et al. [2].

### Factors Related to the Choice of Surgical Specialization

The second finding of the study touches upon the fact that the male gender and a lack of syncopal events in the past medical history constitute factors related to the choice of a surgical specialization. The male gender has a well-substantiated meaning as a factor pertaining to the choice of a surgical specialization by a medical student [8]–[12]. The influence of an experienced syncopal incident on the choice of other than surgical specialization is confirmed by the earlier performed studies at surgeons and instrumenters, which showed that the percentage of the syncopal spells in the medical history of those professionals is significantly lower compared to the general population [13]. The reasons for choosing a specialization are difficult to determine. However, the surgery is characterized by the sight and smell of blood, sharp instruments and longstanding what may discourage vasovagal students.

The third issue elucidated by the study is which syncopal events – outside or inside the operating room or autopsy room – are more important in the choice of surgical specialization. Jamjoom et al. have reported a detrimental effect of syncope or presyncope in the operating room on the choice of surgical specialization [14]. However, those authors did not analyze the influence of previous syncopal events outside the operating room on the choice of the specialization. The results of our study showed that the syncopal and presyncopal events inside the operating room are considered by the students as a more important factor than syncope outside the operating room in the choice of the specialization. However, after taking into account the student’s own assessment that the incident of syncope or presyncope had an impact on the choice of specialization other than surgery, the syncope outside the operating room remained a factor related to the choice of the specialization other than a surgical one.

Paradoxically, women who chose surgical specializations are those without syncopal spells in their past medical history but with presyncopal spells in the operating room. Women who opted for the surgical specialization typically spend a lot of time at the surgical table what increases the risk of presyncopal spells. Otherwise, women not interested in surgical specialization may avoid standing at the operating table what decreases the risk of presyncope.

It seems that experiencing a syncope leads in some students to unconscious resignation from surgical specialization. More studies are needed to establish the link between spontaneous presyncope and syncope characteristics during medical studies, and the level of risk of such events during professional work as a surgeon in following years.

More women attend the medical studies. If this tendency continues, more women will be entering a surgical specialization. Women have lower tolerance of standing position in comparison to men [15], [16] and such a phenomenon was observed in some women during prolonged surgery [13]. More studies are needed to collect a follow-up of such female students. It is plausible that a repeated exposure to upright position could improve their tolerance of prolonged upright position. On the other hand, during periods of such events like menstruation, slight dehydration or fatigue the tolerance of vertical posture could decrease [17], [18].

There is another issue which could be addressed in the future studies of women. It is the influence of prolonged vertical posture on the mother and baby safety during pregnancy in female surgeons.

### Comparison CART and Logistic Regression Analysis Results

The results of CART and the logistic regression analysis were similar but not equivalent. The reason may be related to the lower incidence of syncopal spells in men that in women. The logistic regression analysis did not allow to determine the influence of gender on the choice of surgical specialization because women who had more syncopal episodes also were less prone to choose the surgical specialization.

#### Limitations

The question about the impact of a syncopal or presyncopal event on the choice of a specialization concerned the general effect and not the influence on the decision to resign from the surgical specialization. The statement that syncopal and presyncopal spells discourage students from pursuing a surgical specialization may be a plausible interpretation. However, it is possible that in some cases the students became interested in syncopal spells and cardiology.

The questionnaire only included a question about the age at the syncopal events. It was therefore impossible to perform an analysis of the relation between the time of the last syncopal event and the decision to pursue surgical specialization.

We registered only the number of syncopal spells outside the university not inside. We did not collect the data on the number of presyncopal events. The data regarding medication and blood pressure were not collected what constitutes a limitation of the study. However, the studied population seems to be generally healthy.

Students usually consider more than one specialization and about 80% of them is not really sure which specialization to choose. They rather consider a wide range of possibilities. Therefore, the approach taken in the research is quite close to reality, whereas eliminating the big group of undecided students would lead to an analysis performed only in the subgroup of the most determined students and not the whole population of students.

The population limitation of the study consisted in the fact that only subgroups of students and not the whole population were included in the study. However, the students were chosen randomly what decreases the bias related to the choice of subgroups based on some method (e.g. scientific associations). The statistical limitations are related to the size of the groups, scarcity of syncope during classes at the university and a low proportion of male students in the study caused by the predominance of women at the medical universities.

The models constructed during CART analyses had low sensitivity and specificity, and the accuracy of the global 10-fold cross validation was less than 0.70, suggesting low accuracy of constructed CARTs, However, it could hardly be expected that variables like gender and syncopal history were able to precisely predict such complex decision as the choice of specialization. On the other hand, the non-linear analysis allowed for finding associations which could not be found during linear analysis such as logistic regression.

### Conclusions

1. Syncopal and presyncopal spells influence the choice of the specialization of a medical doctor.
2. Male gender and no history of syncope outside the operating room are the main factors related to the choice of surgical specialization.
3. Women who are interested in the surgical specialization and who did not have syncopal spells outside the operating room are prone to presyncopal events while partaking in the surgeries, what does not discourage them from the surgical specialization.
